# Capture and Release Mechanism of Ni and La Ions via Solid/Liquid Process: Use of Polymer-Modified Clay and Activated Carbons

**DOI:** 10.3390/polym14030485

**Published:** 2022-01-26

**Authors:** Cinzia Cristiani, Maurizio Bellotto, Giovanni Dotelli, Paola Gallo Stampino, Saverio Latorrata, Elisabetta Finocchio

**Affiliations:** 1 Dipartimento di Chimica, Materiali e Ingegneria Chimica “Giulio Natta”, Politecnico di Milano, Piazza Leonardo da Vinci 32, 20133 Milan, Italy; giovanni.dotelli@polimi.it (G.D.); paola.gallo@polimi.it (P.G.S.); saverio.latorrata@polimi.it (S.L.); 2 OPIGEO, SrL, Via dell’Industria 13, 36040 Grisignano di Zocco, Italy; maurizio.bellotto@opigeo.eu; 3 Dipartimento di Ingegneria Civile, Chimica e Ambientale, Università di Genova, Via all’Opera Pia 15, 16145 Genova, Italy

**Keywords:** mixed solutions, polymer modified clay, polymer modified activated carbon, nickel ions, lanthanum ions, capture mechanism, release mechanism

## Abstract

This study is a starting point for the development of an efficient method for rare earths (REs) and transition metals (TMs) recovery from waste electrical and electronic equipment (WEEE) via a hydrometallurgical process. The capture and release capability of mineral clays (STx) and activated carbons (AC), pristine and modified (STx-L6 and AC-L6) with a linear penta-ethylene-hexamine (L6), towards solutions representative of the process, are assessed in the lab-scale. The solids were contacted with synthetic mono- and bi-ionic solutions containing Ni(II) and La(III) in a liquid/solid adsorption process. Contacting experiments were carried out at room temperature for 90 min by fixing a La concentration at 19 mM and varying the Ni one in the range of 19–100 mM. The four solids were able to capture Ni(II) and La(III), both in single- and bi-ionic solutions; however, the presence of the polyamine always results in a large improvement in the capture capability of the pristine sorbents. For all the four solids, capture behaviour is ascribable to an adsorption or ion-sorbent interaction process, because no formation of aquo- and hydroxy-Ni or La can be formed. The polyamine, able to capture Ni ions via coordination, allowed to differentiate ion capture behaviour, thus bypassing the direct competition between Ni and La ions for the capture sites found in the pristine solids. Release values in the 30–100% range were found upon one-step treatment with concentrated HNO_3_ solution. However, also, in this case, different metals recovery was found depending on both the sorbent and the ions, suggesting a possible selective recovery.

## 1. Introduction

Metals are present as a large part of the composition of waste electric and electronic equipment (WEEE). WEEE includes a variety of metal ions, such as heavy metals (HMs), rare earths (REs), precious metals (PMs), and transition metals (TMs). Such a composition poses serious risks to human health and the environment when, at the end of their life, they must be disposed of. Indeed, all these metals can be potentially toxic despite being fundamental to many technological productive sectors, such as electrochemical industries, wood processing, or petroleum refineries [1,2,3].

It is the authors’ opinion that in the 2020–2021 pandemic context, the production and use of various electrical devices, mainly portable, has resulted in the creation of even larger amounts of WEEE. Moreover, E-waste generation and water pollution are only parts of the whole problem; the world technological transition demands for elements (mainly metals) were identified by the EU as critical, thus subjected to a serious supply risk [4,5].

In this respect, an effective WEEE management will contribute to the realisation of the UN’s Sustainable Development Goals and reach the Rome Summit targets (Rome (IT) 30–31 October 2021). The recovery of metal components from WEEE, by implementing a real circular economy, will fulfil the requirements of efficient exploitation of the natural resources, and it will help to reach carbon emission neutrality. For example, precious metals and copper extraction results in CO_2_ emissions, which account for about 1/1000 of the world’s emissions per year [4].

Therefore, European Union countries are asked to take action to ensure technological answers to all these problems, and these technologies have to be targeted not only toward waste treatment or elimination but also to the recovery and reuse of the valuable metals as a secondary raw material [5,6].

More conventional treatment methods (i.e., chemical precipitation, flotation, ion exchange, coagulation/flocculation, adsorption, and electrochemical removal) are already in use, while other unconventional ones (i.e., ultrafiltration, nanofiltration or reverse osmosis) [1,2], have been more recently proposed and implemented in the practice, as reported by D. Fila et al., A. Iqbal et al., and V. Kumar et al. [7,8,9]. However, despite several good efforts on the development of cheap and efficient technologies, in our opinion, there is not a unique solution to the problem. All the proposed methods have, at the same time, significant advantages and disadvantages, adequacies and inadequacies [1,2]. Nonetheless, it is undeniable that, among others, adsorption is one of the simplest and lowest cost methods for pollutants and heavy metal removal in wastewater. Each adsorbent has its own characteristics and adsorption capacity, and large numbers of studies have been focused on developing materials with improved performance and lower costs. For example, improvement of the removal efficiency can be pursued by the modification of shape, size, physical and chemical nature of the adsorbents, as exemplified in several recent reports in the open literature [10,11,12,13,14,15].

Clay-based materials are the most frequently used adsorbent solids due to their structural and morphological properties that meet adsorption requirements [10,16,17,18,19,20]. Moreover, clay materials can be modified by several organic and inorganic treatments to improve their adsorption capacity, as well as their textural structure and surface properties [21,22,23,24,25,26]. Similar considerations apply to carbon-based materials [27,28,29,30,31,32].

In the past, our research group investigated mineral clay (smectite, STx) and carbon-based materials, namely activated carbon (AC) and reduced graphene oxide, both pristine and modified by the functionalization with pentaethylenehexamine (L6) for metal capture and recovery in synthetic solutions simulating WEEE treatment solutions [33,34]. The developed materials demonstrated interesting capture and release capability towards single and mixed solutions containing REs (La, Y, Nd), and Cu, Mn and Fe as representative of TMs. The role of the sorbent nature and composition, and of the operating conditions (e.g., pH, ions concentration, solutions complexity) was tuned for the success of the capture process [35,36]. Adsorption/desorption mechanisms were also assessed [37]. The possible different affinity of each ion for the different solid sorbents, in both mono-ionic and complex matrices, was considered as the determining factor for the processes’ effectiveness [20,22]. The presence of polyamine was found to be fundamental in metal capture and metal release performances.

Considering the typical WEEE composition, Ni also has to be considered, mainly for recovery purposes, being fundamental in technological application [7,9].

The four solid sorbents previously developed in our laboratories, namely pristine and modified mineral clay (STx, STx-L6) and activated carbon (AC, AC-L6), are here proposed as sorbents for a solid/liquid process towards mixed Ni-La solutions.

To the best authors’ knowledge, no studies considering such sorbents systems and solution composition have been reported in the literature. Furthermore, different sorbents’ nature, modifiers, solutions concentration and compositions have been reported and studied [9,18,20,28,32,38,39] Moreover, few papers reported mixed La-Ni solutions [15] or assessed both capture and release mechanisms [8,40,41].

Accordingly, the aim of this paper is to demonstrate the capability of the sorbent solids developed in our laboratories towards metals capture in mono-ionic and bionic La-Ni solutions, with particular attention to capture and release behaviour in the presence or absence of the polyamine. Moreover, it is in the authors’ opinion that the knowledge of the ion-sorbent interaction allows the scaling-up of the sorbent materials and process from the lab-scale conditions to the “in field” ones. Indeed, the knowledge of the involved phenomena can allow for better control of the capture and the related process.

For this purpose, both mono-ionic and bi-ionic solutions were prepared and contacted with the sorbents. Solution compositions were selected considering a pre-industrialization perspective of WEEE treatment via a hydrometallurgical process [38,39,42,43], and also for the sake of comparison, considering the composition of those previously analysed and reported. Both the capture and release processes were performed according to the best previously studied conditions to maximise the performance of the total process [35,44]. A preliminary description of both capture and release mechanisms is attempted on the basis of the solutions analysis only.

Therefore, this study is intended as a step forward towards the development of an efficient approach for rare earths (REs) and transition metals (TMs) recovery from waste electrical and electronic equipment (WEEE) via hydrometallurgical processes and using natural and low-cost sorbent solids.

## 2. Materials and Methods

### 2.1. Sorbent Solids Characteristics

Standard Ca-montmorillonite STx-1b (STx in the following, supplied by Clay Minerals Society, Gonzales County, TX, USA), of formula ^IV^Si_4.0_^VI^(Al_1.21_Fe^3+^_0.05_Mg^2+^_0.36_Ti_0.02_)^XII^ (Ca_0.14_Na_0.02_K_0.01_)O_10_(OH)_2_ and powdered activated carbon (AC in the following, supplied by Torchiani s.r.l., Brescia, Italy) were used as sorbent materials.

The purity of the selected clay mineral is certified by the supplier, and reported in the technical data sheet with the following composition: 95–100% Montmorillonite (CAS-No.1302-78-9 95) and 1–5% Quartz (CAS-No.14808-60-7) [45]. Therefore, adsorption properties can be considered due to the montmorillonite component only.

Morphological characteristics of the pristine sorbent solids are reported in Table 1.

Both clay and carbons were modified by intercalation of a linear penta-ethylene-hexamine, of formula C_10_H_28_N_6_, (L6 in the following, supplied by Sigma Aldrich, St. Louis, MO, USA. 99% pure). The intercalation process was performed according to literature reports [35,44].

It has been demonstrated that the experimental conditions applied in the intercalation reaction allow for the polyamine intercalation in its neutral form, i.e., without any ion exchange and no formation of ammonium salts. This result was confirmed by the absence of Ca ions released in the solution after intercalation [44].

This way, both the CEC value of the clay (i.e., 1.24 mmol/g) and the polyamine coordination capability of the amino-groups towards metal ions were preserved [44].

The final polyamine content in both organoclay (STxL6 in the following) and organocarbon (ACL6 in the following) was set at 0.40 mmol/g_sorbent_.

All four solids, i.e., Stx, StxL6, AC and ACL6, were tested in the capture and release experiments.

### 2.2. Capture and Release Experiments

For the capture process, mono-ionic and bionic solutions were prepared, starting from nickel nitrate Ni(NO_3_)_2_·6H_2_O 98.5%, and lanthanum nitrate La(NO_3_)_3_·5H_2_O 99.99% (both from Sigma Aldrich, Milan, Italy); other chemicals were HNO_3_ (ACS 70%, Sigma Aldrich, Milan, Italy), and deionized water.

Both mono-ionic and bionic solutions were prepared and contacted with the four solid sorbents. Mono-ionic solutions were prepared at 19 mmol/L. Contacting experiments with bionic solutions were carried out by fixing the lanthanum concentration at 19 mmol/L, and varying the nickel one at 19, 50 and 100 mmol/L, respectively. This way, solutions with initial Ni/La ratios equal to 1, 2.6 and 5.3 were obtained. The molar and weight composition of the initial solutions are reported in Table 2.

Compositions were selected to be representative of Ni and La content in leaching solutions from WEEE treatment and to be comparable with previous results on different metal ions [35].

In a typical capture experiment, 2 g of pristine or modified sorbent were contacted with 50 mL of solution, under stirring at 25 °C for 90 min. During the experiments, the pH was measured by the Mettler Toledo FE20/EL20 digital pH-meter (Mettler Toledo, Milan, Italy), but no pH correction was applied. For all the experiments, the pH was in the range 5–5.5 for clay-based systems and 5.5–6 for carbon-based ones.

One step release process was performed by treating 2 g of the used sorbent with 50 mL of an HNO_3_ solution at pH = 1, under stirring at 25 °C for 90 min. At the end of the 90 min, the reaction was stopped and the solid-liquid separation was performed. No intermediate evaluation of the metal release was done. It has been demonstrated that these conditions are the most proper to reach the maximum metal release without any polyamine release when present [35,44].

Considering the experimental conditions of both capture and release, no changes in the ions oxidation states can occur. Therefore, for the sake of simplicity, La and Ni ions along the text will be indicated sometimes without their charge; however, they have always been intended as Ni^2+^ and La^3+^.

After both capture and release reaction, solid and liquid phases were separated by centrifugation at 13,000 rpm for 1 h (HETTICH 32 RotoFix centrifuge, Hettich Italia, Milan, Italy). From these conditions, total solid-liquid separation is obtained, confirmed by the formation of a complete clarified supernatant solution.

All the solutions were analysed by inductively coupled plasma optical emission spectrometry (ICP–OES) analyses, using a Perkin Elmer Optima 2000DV spectrometer (Labx, Midland, ON, Canada). For all the analyses, the average of three measurements is reported, and the measurement error estimated from the replicate measurements is within 1%. Ion determination was performed considering interferences when present.

Captured metals were determined by the difference between the ions content in solution before and after the capture process; while the metal release was determined by the difference between the metal content in the solids after capture and in solution after release.

Ions behaviour in solution was checked by MEDUSA^®^ software calculation (version 16.1), Figure S1.

### 2.3. Characterization

Solids were analysed by different physico-chemical analyses:

- Laser Granulometry by means of CILAS 1180 (dispersion in water, CILAS, Orléans, France), according to the technical report [46].
- BET analysis (N_2_ as analysis adsorptive, bath temperature 77.35 K), while the porosity of the material was determined by Hg intrusion by means of Autopore V9600 (Micromeritics Instrument Corporation, Norcross, GA, USA). Samples were degassed overnight at 60 °C, (heating rate from 25 °C to 60 °C 1 °C/min).
- X-ray powder diffraction (XRD) patterns were recorded with a Bruker D8 Advance diffractometer (Bruker Italia, Milan, Italy) using a graphite-monochromated Cu Kα radiation, 2θ range 2–65°, step scan 0.02° 2θ and 1 s per step.
- Thermal analyses (TG-DTG) were performed with a DTA-TG SEIKO 6300 thermal analyzer (SEIKO, Riga, Latvia), flowing air, temperature range 25–1000 °C, and heating rate 10 °C/min.

## 3. Results

### 3.1. Clay-Based Sorbent Solids

#### 3.1.1. Metal Capture

Metal capture was studied on both unmodified (STx) and modified (STxL6) sorbents, and metal adsorption capability was tested on both single and mixed ions solutions (compositions according to Table 2). When the single ion solutions were applied, metal content was fixed at 0.475 mmol/g_sorbent_; while for the mixed solutions, La content was kept constant at 0.475 mmol/g_sorbent_, and Ni was increased up to 2.5 mmol/g_sorbent_.

**Figure 1:**
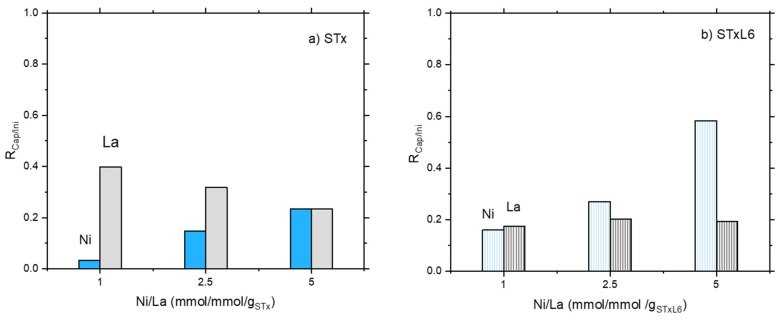
The ratio between metal capture and contacted metal as a function of Ni/La ratio in the initial solution: (**a**) unmodified STx and (**b**) modified STxL6.

In Figure 1a,b the ratio R_Cap/Ini_ equal to (Captured Metal ion/Initial Metal ion) (mmol/g_sorbent_) is plotted as a function of the initial Ni/La (mmol/mmol/g_sorbent_).

**Figure 2:**
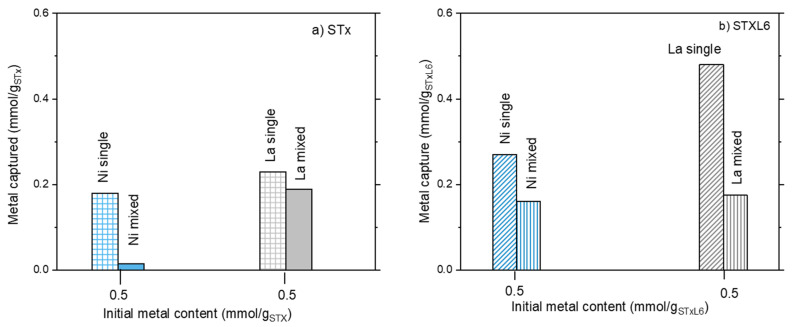
The ratio between metal capture and contacted metal at the same initial ion concentration in single and mixed metal ions solutions: (**a**) unmodified STx and (**b**) modified STXL6.

Considering mixed solutions, the same behavior was found for Ni adsorption for both systems; Ni capture increased on increasing Ni content in the initial solution (Figure 1a,b). However, modified STxL6 was always able to uptake larger amounts of Ni ions (Figure 1b). Different is the case of La capture, where a certain effect of the sorbent nature appeared to be present. Furthermore, La capture lowers on increasing the initial Ni/La ratio with pristine STx, while it is almost constant in the case of STxL6. Considering that contacted La was constant, it can be hypothesized that a site competition between Ni and La is manifest for STx (Figure 1a). To verify this hypothesis, we performed the comparison of the capture capability of both STx and STxL6 with solutions containing only Ni or La of a concentration fixed at 0.475 mmol/g_sorbent_, with a mixed solution containing both ions at the same concentration of the single one. Results are plotted in Figure 2a,b.

It is evident that the metal capture in the single ion solutions was always higher than in the mixed ones, confirming that ions are competing for the same capture sites. This behavior is apparently insensitive to the sorbent composition, suggesting that La and Ni, qualitatively, interact with the sorbents via similar mechanisms.

#### 3.1.2. Metal Release

**Figure 3:**
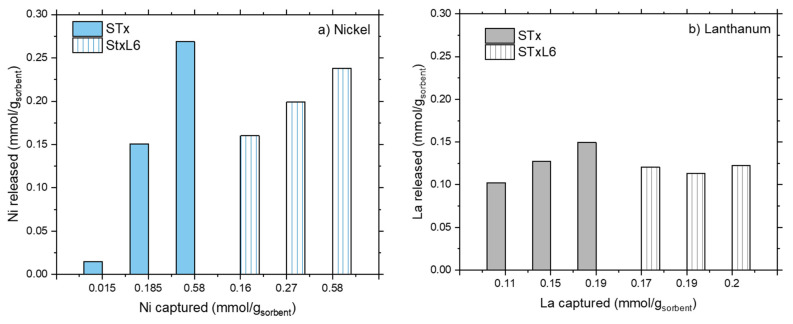
Metal release as a function of the metal captured for STx and STxL6.

The release unit operation was performed according to the experimental conditions described in the Materials and Methods section, thus solids after capture reaction were treated by a nitric solution at pH = 1. Release results are plotted in Figure 3a,b.

For both the sorbent solids, considerable amounts of captured ions are released, i.e., from 30 up to 100% of the captured ones, when treated at pH = 1. However, different behaviors were found depending on the ion. Let’s start by considering Ni ions (Figure 3a). For both STx and STxL6, Ni release is only a function of captured Ni; larger absolute release corresponds to the larger capture. However, the 100% of release was obtained only for the lowest capture value (i.e., 0.015 and 0.16 mmol/g_sorbent_, for STx and STxL6, respectively), then release percentages progressively decreased down to 40–45% in both cases. Furthermore, in the presence of the polyamine, the fraction of Ni released was always lower than in the pristine clay.

This differs in the case of La ions where La release was always in the range of 80–100% for STx, and 60–70% for the modified STxL6.

The difficulty in obtaining a total metal release suggests the presence of a relatively strong metal-sorbent interaction, as it could result from an exchanging or a coordination process. Moreover, considering the similar behavior of STx and STxL6 towards Ni and La, both capture mechanisms (i.e., ion exchange and ion coordination) can be hypothesized.

The incomplete release, possibly related to different metal-sorbent interaction strength, apparently suggests lower effectiveness of the release process, which could prevent total Ni and La recovery. However, such a result has been obtained in a “one-step” release only; therefore, a multistep process (for instance, subsequent treatment at pH 1) could allow for the total metal recovery, as already reported in the literature for La ions [37].

#### 3.1.3. Solid Characterization

**Figure 4:**
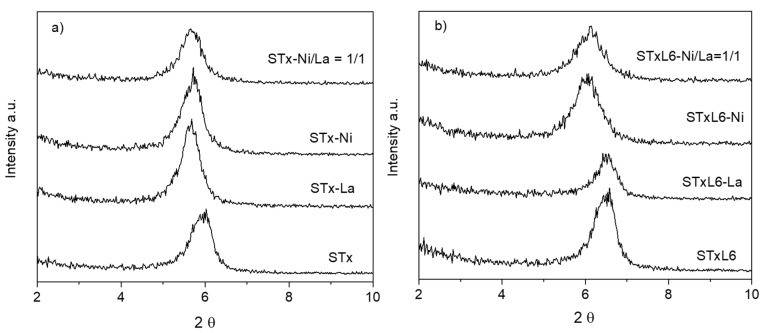
XRD patterns of the sorbent solids after the capture of La and Ni in single ion and mixed Ni/La solutions: (**a**) pristine STx and (**b**) modified STxL6 (STx and STxL6 are reported for comparison).

Solids after the capture step were characterized by XRD; patterns are plotted in Figure 4a,b, where, for the sake of clarity, only the range of the basal reflection (d_001_) is reported. Regarding clay-based materials, the analysis of the basal reflection displacement has been demonstrated to be highly informative about interlayer occupancy [44,47,48,49,50,51,52].

For the sake of completion, spectra in the full range of collection (2–65 2θ°) are reported in the Supporting Information (Figure S2), namely pristine STx and Stx treated with a nitric acid solution at pH = 1, i.e., the release conditions. The relatively low noise in the XRD patterns is essentially due to the microcrystalline nature of the clay, distributing the diffracted intensity in a broad angular range.

When La and Ni are captured by STx, a displacement of the basal reflection towards lower angles, i.e., higher d_001_, was observed, apparently irrespective of the ion nature. The same displacement was also maintained when Ni and La ions were co-captured by the solid, once more suggesting that the interlayer modification was independent of the ion nature and content. It has to be reminded that, for the mixed composition discussed here, the absolute value of captured La was largely higher than Ni, 0.189 mmol_La_/g_STx_ and 0.015 mmol_Ni_/g_STx_, respectively. However, the co-presence of Ni and La ions in the interlayer, indicated by the broadening of the reflection, might be a result of a progressive disordering of the interlayers, being occupied by ions of different dimensions and/or water molecules coordination.

In the case of STxL6, the displacement of the basal reflection, corresponding to a d_001_ enlargement, was observed only in the presence of Ni ions. Similarly to STx, a broadening of the basal reflection was manifest in the co-presence of captured Ni and La, which could suggest again the presence of interlayers randomly occupied and/or different capture sites.

**Figure 5:**
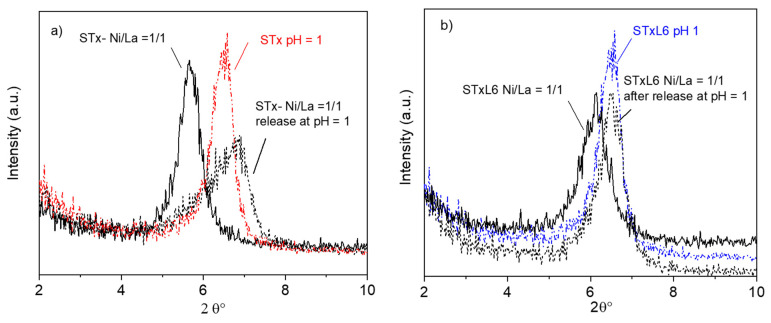
XRD patterns of sorbent solids after release for the mixed Ni/La systems: (**a**) pristine STx and (**b**) modified STxL6 (STx and STxL6 treated at pH = 1 and samples after the capture step are reported for comparison).

XRD analysis was also performed on the solids after the release step; diffractograms are reported in Figure 5a,b (scale is magnified compared to Figure 4). For the sake of comparison, in the figure, the patterns of the solids before release are reported, together with the diffractograms of STx and STxL6 treated at pH = 1, i.e., the same pH applied in the release unit operation.

Upon release, the maximum of the basal reflection was displaced towards higher angles in both sorbent systems, indicating that shrinkage of the interlayer has occurred, possibly due to the removal of the captured cations; as a matter of fact, the maximum position after release was near to the unused sorbent solid treated at pH = 1. The largest maximum displacement was observed for the unmodified STx system, where the basal reflection position was shifted by 1.5° 2θ (compare black continuous and black dotted lines in Figure 5a). Moreover, upon the release step, in the STx diffractogram, a broadening and a marked loss of symmetry of the basal reflection was manifest. All these pieces of evidence are again in line with the presence of a heterogeneous capture sites situation, which reflects on the sorbent rearrangement after capture and release. All these phenomena are limited in the STx-L6 system, where only a little asymmetry, i.e., the shoulder on the left side of the maximum, was manifest. Furthermore, in this case, protons of the nitric solution primarily interact with the polyamine.

### 3.2. Activated Carbons-Based Sorbent Solids

#### 3.2.1. Metal Capture

**Figure 6:**
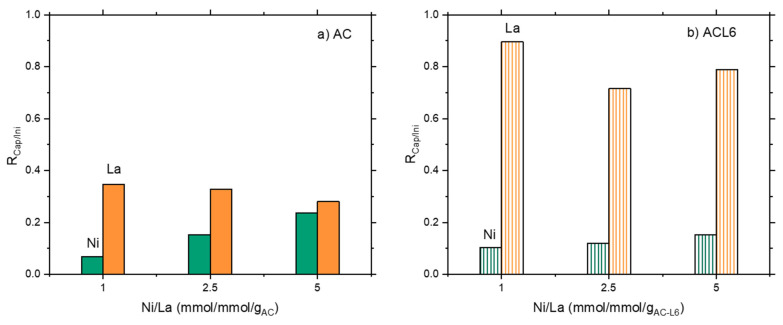
The ratio between metal capture and contacted metal as a function of Ni/La ratio in the initial solution: (**a**) unmodified AC and (**b**) modified ACL6.

**Figure 7:**
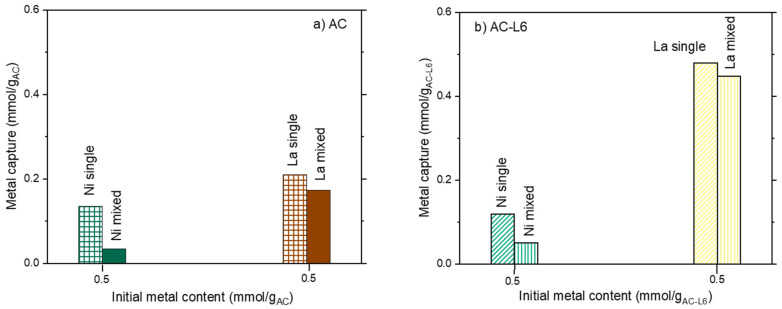
Comparison of the ratio between metal capture and contacted metal in La and Ni single ion and Ni/La mixed solutions for (**a**) unmodified AC and (**b**) modified ACL6.

Additionally, the use of activated carbons, both pristine (AC) and polymer-modified (ACL6) was tested in single ions and bi-ionic solution for Ni and La capture. Activated carbons, indeed, are widely applied in industrial practice for sorption purposes [53]. Results are plotted in Figure 6 and Figure 7a,b, where the ratio R_Cap/Ini_ equal to [Captured Metal ion/Initial Metal ion] (mmol/g_sorbent_) is plotted as a function of initial Ni/La (mmol/mmol/g_sorbent_).

In bi-ionic solutions, when pristine activated carbons were used, Ni capture increased on increasing initial Ni content in solution, showing a positive trend similar to that observed for the clay materials (Figure 6a). However, polymer modification did not improve Ni adsorption over ACL6, which was constant or even lower than unmodified AC (Figure 6b). The La capture was largely higher than Ni on AC and even three times larger when ACL6 is used. However, capture is slightly decreasing in presence of both AC and ACL6.

The effect of the ions co-presence was investigated too; a comparison of adsorption in single- and multi-ions solutions are plotted in Figure 7a,b.

A marked decrease in Ni capture was found in presence of La for both AC and ACL6 sorbents. Ni capture was halved in the case of ACL6 (Figure 7b) and even reduced to one third in the case of AC (Figure 7a). Additionally, La capture was lowered when in a mixed solution (Figure 7a,b). However, the La decrease was relatively low compared to Ni, and such evidence once more suggests a Ni and La competition for capture sites.

#### 3.2.2. Metal Release

**Figure 8:**
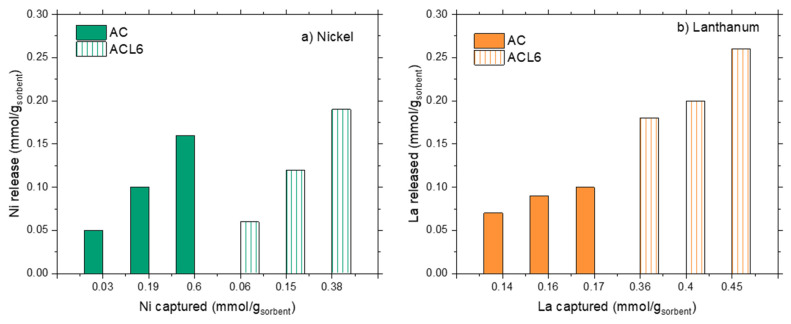
Metal release as a function of the metal capture for AC and ACL6.

Metal release upon treatment with HNO_3_ at pH = 1 is plotted in Figure 8a,b, for nickel and lanthanum, respectively. Irrespective of the sorbent nature, Ni and La presented the same behavior—absolute metal release increased on increasing the absolute ions capture. However, from a quantitative point of view, a marked difference can be found between the released amount of the two ions.

When AC is used, Ni release decreased from 100% down to 26%, respectively, for the lowest (0.03 mmol/g_AC_) and the highest (0.6 mmol/g_AC_) Ni content in the solid; while La release was almost constant in the range 53–56%. Similar considerations can be done for the absolute release in the ACL6 system; the Ni release decreased from 80% down to 50% on increasing the metal loading in the solid, while La release was almost constant at 52–56% of the total captured metal.

## 4. Discussion

As reported in the literature, [18,19,20,27,28], both clay-based and carbon-based systems can be effective sorbents for metal ions, in particular, Ni and La. In accordance with the literature, our four systems have demonstrated good sorption capability for the different ions concentrations in both single- and bi-ionic solutions.

### 4.1. Capture Process

From the experimental data, the ions capture is apparently exerted by the solids via different mechanisms that might depend on the sorbent nature and the presence of the polyamine. Therefore, the capture extent is mainly related to the ion nature, which ultimately can be traced back to ion-sorbent interaction.

**Figure 9:**
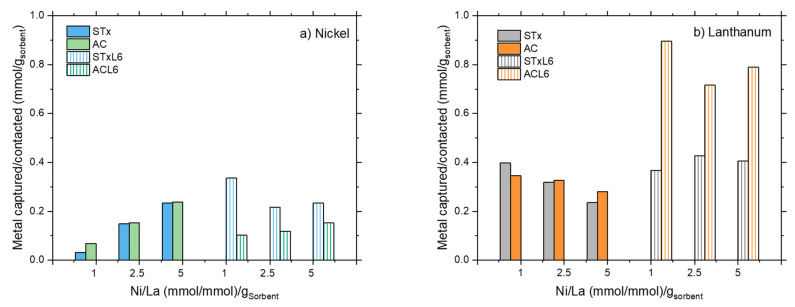
Comparison of the capture capability of the different sorbents towards.

To better analyze this point, in Figure 9 the comparison of the capture extent by the four sorbents are summarized for Ni (Figure 9a) and La (Figure 9b).

Regarding Ni, the capture capabilities of the pristine solids (STx and AC) are comparable; they are able to capture Ni in both mono- and bionic solutions, in increasing amounts on increasing the Ni content in the initial solution. In STx, capture is mainly due to ions exchange with interlayer cations, the typical mechanism in layered clay minerals, such as montmorillonite STx-1b. Indeed, the exchange mechanism is still present and active upon strong solid modification, as exemplified in the literature [25].

In general, the polyamine slightly improves the capture performance in the case of the clay-based materials, while polyamine-modification worsens the performance of carbon-based ones (Figure 9a).

However, all the sorbents are more prone to La capture, suggesting a larger affinity of the pristine materials for La, which is further increased upon modification. In conclusion, the extent of La capture is always higher than the Ni one, irrespective of the sorbent’s nature. The observed different capture capability can be related to the two capture mechanisms in these sorbents, i.e., ion exchange due to the mobility of Ca ions in the interlayer and coordination by the polyamine, when present. These mechanisms are always present, but become active depending on the affinity of the different ions for the clay or the polymer.

To have a deep insight on this point, the use of chemical analysis can be helpful; indeed, the ionic exchange can be directly measured by the evaluation of the Ca ions present in the solution after the contacting step. Accordingly, the charge balance (i.e., number of charges out and number of charges in) must correspond to maintain electroneutrality. Results (by ICP analysis) for Ni and La capture in single-ion and mixed solutions with equimolar ratio (Ni/La = 1) are compared in Table 3.

Let’s consider pristine clay first. It is evident that Ni capture occurs via the ions exchange mechanism, as the charge balance is preserved by the total Ni ions “in” (0.36 mmol/g_STx_) and the total Ca ions “out” (0.32 mmol/g_STx_), (Table 3).

When La is co-present in the solution, sites competition occurs, so that Ni capture drops down to a very limited extent (0.015 mmol/g_STx_). Despite the different charge, La ions better interact with the clay and are able to displace Ca ones better than Ni. This behavior could be explained by considering the dimensions of La ions (1.06–1.216 Å), which on average are close and even slightly bigger than those of the exchangeable Ca ones (0.99–1.12 Å) [54,55]. Accordingly, the total exchanged charge, 0.5 mmol/g_STx_, accounts for the total Ni and La capture (0.6 mmol/g_STx_, Table 3).

This picture is also supported by XRD analysis, where a broadening of the basal reflection is manifest when La and Ni are co-captured. Thus, the final arrangement of the sorbent solid, after capture, results from a random occupation of the interlayer positions by the different ions replacing the Ca ones.

Ni capture is improved in the presence of the polyamine (0.27 mmol/g_STx_ vs. 0.18 mmol/g_STxL6_, for STxL6 and STx, respectively). It has been reported in the literature [36,43] that, in the case of mixed solutions containing a RE and a TMs ion, namely La(III) and Cu(II), capture reaction occurs via a mixed mechanism. Indeed, La is mainly exchanged with Ca, while Cu is mainly coordinated by the polyamine. The observed increase of Ni capture might be due to the small dimensions of Ni(II) ions and hydrated Ni(II) ions (0.69 and 4.04 Å, respectively [54]), which might improve Ni mobility, thus favoring the interaction with an increased number of sites of the polyamine chain. Moreover, we can speculate the interlayer contraction due to the polyamine intercalation may favor Ni exchange.

In presence of La, as already observed for pristine STx, in STxL6 Ni, capture decreases as well (Table 3). However, La capture in pristine and modified STx showed very close values (0.19 mmol/g_STx_ and 0.175 mmol/g_STxL6_ for STx and STxL6, respectively), with the total La charge always accounting for the total exchanged one (Table 3). Accordingly, in STxL6, the exchanging mechanism involving Ni ions is clearly hampered by La competition. However, the capture value remains high, and at least comparable with that of unmodified STx, confirming the co-existence of both mechanisms.

Accordingly, when STxL6 is used, La ions are still mainly captured via the exchange mechanism; while Ni is captured via coordination. Such a picture is in line with our previous reports for similar systems [37,43]. The absence of the basal shift can be traced again to a random occupation of the interlayers.

La affinity for the clay and its capability to replace Ca ions have been already reported in the literature [47], both for single- and mixed ions solutions [43]. Peculiar is the Ni ions, which, despite close chemical similarity with Cu ions, show a completely different behavior. It has been reported in the literature [43] that, in the case of Cu, the coordination with the polyamine was the only possibility for Cu ion capture; no exchange was possible in any case, neither in mixed- nor in single-ion solutions. An explanation for such a difference was traced back not only to the ions dimension but also to the coordination bond strength and the ions capability to form aquo-complexes in solution before/during the capture step.

The fields of the existence of the different nickel species, in single- and mixed-ion solutions, as a function of pH, were evaluated by MEDUSA^®^ software (results in Supporting Materials Figure S1). Ni (II) ions are stable species at a pH lower than pH = 5–5.5 (i.e., the operating pH of this work); therefore, no formation of Ni aquo-complexes is expected, either in single-ion solution or in the mixed one. These data are in line with literature indications, which reports for Ni a minor tendency to form aquo-complexes with respect to Cu [43,56]. Thus, the exchanging mechanism, in this case, is not prevented, and Ni capture can occur in STx as well.

This point is of paramount importance for future in-field applications, because the different behaviors exerted by ions of similar chemical nature, towards the same sorbent solid, could pose a base for selective capture in complex solutions, as in the case of the real practice.

Moreover, considering that ΔCharge—calculated by the difference of displaced Ca ions and total captured Ni and La—is always negative, additional adsorption by the clay surface, for instance, can be hypothesized according to the literature [57]. Indeed, for Ni ions capture by the use of clay-minerals, Mo et al. recently [57] have suggested, together with surface complexation, a precipitation process, due to a ‘‘continuous nucleation” mechanism induced by the sorbent surface. Near adsorption sites saturation, a similar effect may be active in the case of both Ni and La ions.

Carbon-based materials (Figure 9b) are more efficient in La capture, the largest ion; therefore, no diffusion limitation may be present in the matrix pores. Carbon modification by the polyamine does not result in increased Ni capture, and apparently, the coordination mechanism is not as relevant as it is for the modified clays. This could be due to the nature of pristine active carbons’ surface. It is well established that in carbon-based materials, adsorption is the active mechanism for heavy metals capture [32]. Capture is mainly dependent on a sorbent material’s specific surface area, pore size, and pore volume, but also the nature of the surface, i.e., the surface functional groups play a fundamental role in the process [27,41]. Indeed, the surface chemistry of carbon-based materials presents a degree of heterogeneity associated with heteroatoms coming from the nature of the AC precursors or inoculation methods applied during activation [58].

Such modifiers, interacting with the nitrogen electron lone pairs, could partially affect the availability of the polyamine nitrogen atoms for the metal coordination, thus affecting capture performances.

Regarding La capture by ACL6, it is evident that the polyamine markedly increases La capture; however, it may not be due to a direct La-polyamine interaction only.

In this respect, it has to be reminded that, also for these materials, different ions-sorbent interactions may be present; they may include electrostatic interactions, π-π electronic donor-acceptor interactions, hydrogen bonding, ligand exchange, pore filling, ion exchange, surface complexation, and hydrophobic interactions [27], but ions exchange and partial hydrolysis [41], too. These interactions are pH-dependent, but they can also be contemporarily active.

To clarify this point, MEDUSA^®^ calculation for La ions (Supporting Information, Figure S1a) was performed. On the basis of the calculation, the formation of La hydroxides is expected due to pH. Therefore, lanthanum behaviour may be related to the more alkaline environment generated by the presence, at the surface of the activated carbon for both the original modifier groups and the polyamine. Accordingly, the larger La capture could be accounted for by additional La hydroxides precipitation near the surface, induced by the surface itself.

### 4.2. Release Process

For practical application, metal release is also important. No or negligible metal release is allowed when the sorption process is thought of as definitive metal trapping for landfill confinement; on the contrary, an easy and possibly total release is asked when the sorption process is targeted to metal recovery and re-use.

On the basis of previous literature indications [15], metal release at very low pH (pH = 1) has been experienced. In the authors’ opinion, harsh conditions can provide information on ion-sorbent interaction strength that is useful enough to satisfy both landfill and recovery applications. Moreover, low pH is reported as the best condition to promote quantitative desorption [8,41].

**Figure 10:**
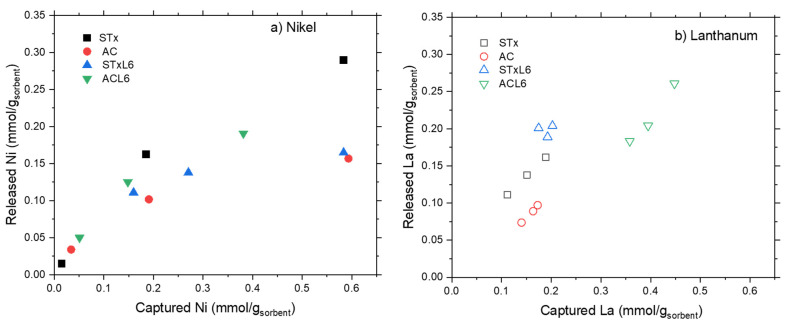
Comparison of metal release for the four sorbents.

Trends of metal release, after one step, are plotted in Figure 10a,b, while relative percentages are summarized in Table 4.

Nickel release evidences a threshold value that prevents total release at high Ni capture (Figure 10a), while lanthanum release can be considered almost linear. Additionally, in this case, total release can be reached only at low La capture (Figure 10b). The incomplete metal release, after one-step treatment, pinpoints the presence of heterogeneous capture sites characterized by different interaction strengths. Some of them are strong enough to stably and safely trap the ions, and, despite the harsh release conditions (pH = 1), they probably need a multistep process for the total recovery of the metals. Some others are more weakly interacting with the ions, thus more prone to metal release.

From a quantitative point of view, an almost constant lanthanum release is achieved in any case. The release is very high in the case of the clay-based materials (80–100% of total La), but considerably lower for the carbon-based ones (50–58%) (Table 4).

Regarding Ni, a direct comparison can be done between the samples of STx and STxL6 with the same capture, (i.e., 0.58 mmol/g_Stx_,). Ni release of 50% and 67% is obtained for STx and STxL6, respectively, while for the carbon-based sorbents this behavior is even more pronounced, considering that a Ni release of 26% is reached for pristine active carbon, while a release of 50% for ACL6.

It has to be reminded that in these systems when the ion-sorbent system is contacted with a solution at pH = 1, protons, smaller and faster, displace metal ions in both the interlayer (via exchange mechanism) and polyamine (via protonation of the amino-groups); this results in the metal release. Accordingly, the larger nickel release in the polymer-modified materials could be due to Ni displacement by protons in both exchanging and coordination sites.

More complex is the lanthanum situation, where the total release is observed in clay-based materials only, irrespective of whether or not the polyamine is present. In this case, a total replacement of La ions by protons in the interlayer has to be hypothesized, with La occupying the original Ca interlayer position.

Ni and La replacement by protons are also supported by the evaluation of d_001_ after the release process, reference being made to the d_001_ values corresponding to those measured for the sorbent solids when treated at pH = 1, i.e., 12.5 Å for STx and 13.7 Å for STxL6.

In the case of the carbon-based sorbent, the low and constant release (about 50% of the total capture) is the result of the sites’ heterogeneity discussed above. La ions bonded to sites of different strength cannot be totally recovered by a one-step process. It can be speculated that released La ions correspond to those more weakly interacting, for instance via electrostatic interactions, surface complexation, or hydroxides formation [27]; thus, are more prone to pH etching or dissolution.

As a concluding remark, all this evidence may suggest a possible effective and selective treatment of mixed and complex solutions, so as to lay the foundations for realistic industrial development of both the materials and the process.

Regarding the mechanisms undergoing the capture and release process, this paper was able to give only preliminary information on them. Indeed, to better clarify all the aspects related to the ions-sorbents interaction, a full and deeper solids characterization after their use is needed. For instance, the use of spectroscopic and microscopic techniques that are able to “read” the surface morphology and/or magnify the ion-solids interaction is thought to be very helpful to get the point.

## 5. Conclusions

In this study, polymer-modified clay and active carbon have been proven to be effective solid sorbents toward Ni ions and La ions in mono- and bi-metallic aqueous solutions. Modification by L6 polyamine only slightly increases the amount of adsorbed Ni ions from bi-ionic solutions in comparison to pristine clay, possibly by improving the coordination mechanism, while the same effect was not detected over modified active carbons. The capture of La ions, however, is significantly higher in modified clay. Different ion-sorbent interactions are possible: ions exchange, surface adsorption, due to the clay, and coordination due to the intercalated polyamine.

Both lanthanum and nickel are involved in the exchange process since they are both captured by pristine clays. However, Ni is preferentially coordinated to the polyamine, in view of its interaction with amino groups.

The observed release in the 30–100% range upon the one-step process is in line with the presence of a heterogeneous capture sites’ distribution. Such heterogeneity, indeed, leads to different solid/ions interaction strength, i.e., some sites are strong enough to stably trap the ions, while some others are less interacting, and thus more prone to metal release.

## 6. Final Considerations and Future Perspective

The results reported here are the last of a series already published on this topic, and consider these four solids, namely STx, Stx-L6, AC, AC-L6, as possible sorbents for capture and recovery of metals from WEEE.

It is undeniable that the four proposed solids, in view of the polyfunctionality of their sites, are able to capture and release a variety of TMs (Ni, Cu, Mn, Fe) and REs (Y, La, Nd), not only in single-ions solution but also in mixed ones.

In mixed solutions, despite acceptable capture and release percentages, reaching for the whole process efficiency of 80–90% at least, there is still a low selectivity present, when only one solid is thought of as the choice for the process. In this paper, however, it has been demonstrated that the polyamine, able to capture Ni ions via coordination, allowed to differentiate ion capture behaviour, thus bypassing the direct competition between Ni and La ions for the capture sites found in the pristine solids. Similar findings were also found in the case of the release process, where different metal recovery occurred depending on both the sorbent and the ions nature. Accordingly, this points toward possible selective recovery and reuse of the metal ions to be used as secondary raw materials. Therefore, it is the authors’ opinion that a few points have to be considered for an effective sorbent process using the following proposed solids:

1. The combination sorbent-ion is fundamental. There is no unique sorbent selection, and due to the system complexity, more than one sorbent may be considered;
2. Therefore, the most proper sorbent has to be selected in light of the solution composition to be treated and of the chemical behavior of the ions in presence of the sorbent;
3. Possibly, the smartest approach to a real process design should imply the presence of an in series multistep process where several treatment units filled with different solid sorbents are applied, and selective ions separations are performed step by step.

On this basis, this study, together with previously reported studies, lays the groundwork for the development of an efficient method for rare earths (REs) and transition metals (TMs) recovery from WEEE via hydrometallurgical processes.

## Figures and Tables

**Table 1:** Physico-chemical properties of pristine clay (STx) and activated carbon (AC).

Sorbent Solid	Physico-Chemical Properties
STx	Particle size = 20 ± 10 µm
	Specific Surface area (SSA) = 86.5 m^2^/g
	Specific Pore volume (Vp) = 0.35 cm^3^/g
	Pores dimensions (dp)= 77 and 140 Å
	Cation Exchange Capacity (CEC) = 1.24 mmol of charges/g
AC	Particle size = 99.8% < 120 μm
	Specific Surface area (SSA) = 575 m^2^/g
	Humidity percentage = 10%
	Ash = 10–15%
	Density 600 kg/m^3^

**Table 2:** Initial composition of the solutions applied in the capture experiments (average of 3 measurements estimated error from the replicate measurements is within 1%).

[Ni] mM	[La] mM	Ni/La	Ni (mmol/g_sorbent_)	Ni (mg/g_sorbent_)	La (mmol/g_sorbent_)	La (mg/g_sorbent_)
19	19	1	0.475	27.9	0.475	66.02
50		2.6	1.25	73.4		
100		5.3	2.5	146.7		

**Table 3:** Charge balance calculation upon capture step for mono-ionic and bi-ionic solutions in presence of STx and STxL6 sorbent solids. (* ΔCharge = ∑Charge_out_ − ∑Charge_in_) where Charge_in_ and Charge_out_ include the different ionic species. (Average of three measurements, error estimated from the replicate measurements is within 1%).

Sorbent	Solution Composition (mmol_ion_/g)	Ni_captured_ (mmol/g)	La_captured_ (mmol/g)	Ca_released_ (mmol/g)	Charge_in_ Ni^2+^ (mmol/g)	Charge_in_ La^3+^ (mmol/g)	∑Charge_in_ (Ni^2+^+La^3+^) (mmol/g)	∑Charge_out_ Ca^2+^ (mmol/g)	ΔCharge * (mmol/g)
**STx**	Ni = 0.475	0.18		0.16	0.36			0.32	−0.04
	Ni/La = 0.475/0.475	0.015	0.19	0.25	0.03	0.57	0.6	0.5	−0.1
**STxL6**	Ni = 0.475	0.27		0.24	0.54			0.48	−0.06
	Ni/La = 0.475/0.475	0.16	0.175	0.24	0.32	0.52	0.85	0.49	−0.36

**Table 4:** Comparison of metal release percentages as a function of the absolute capture values for the four different sorbent solids.

Nickel							
STx		STxL6		AC		ACL6	
Capture mmol/g	Release %	Capture mmol/g	Release %	Capture mmol/g	Release %	Capture mmol/g	Release %
0.015	100	0.16	100	0.034	100	0.05	100
0.185	88	0.27	100	0.19	53	0.15	84
0.58	50	0.58	67	0.59	26	0.38	50
**Lanthanum**							
**STx**		**STxL6**		**AC**		**ACL6**	
**Capture** **mmol/g**	**Release** **%**	**Capture** **mmol/g**	**Release** **%**	**Capture** **mmol/g**	**Release** **%**	**Capture** **mmol/g**	**Release** **%**
0.11	99	0.175	100	0.14	53	0.36	51
0.15	91	0.19	100	0.165	54	0.39	52
0.19	86	0.20	98	0.17	56	0.45	58

## Data Availability

All data are available upon reasonable request from the corresponding authors.

## Supplementary Materials

The following supporting information can be downloaded at: https://www.mdpi.com/article/10.3390/polym14030485/s1. Figure S1: MEDUSA^®^ Plots for Ni, La and mixed Ni/La solutions; Figure S2: XRD pattern of pristine STx and Stx treated with nitric acid solution at pH = 1, full spectral range.
