# Supplementary material for: Capture and Release Mechanism of Ni and La Ions via Solid/Liquid Process: Use of Polymer-Modified Clay and Activated Carbons
Source: Polymers (Basel). 2022 Jan 26;14(3):485. doi: 10.3390/polym14030485 (PMC8840614; doi:10.3390/polym14030485)
Supplement: Supplementary file 1 [file polymers-14-00485-s001.zip › polymers-1503420-supplementary.pdf]

*polymers*

MDPI

# Supplementary Information

# Capture and Release Mechanism of Ni and La Ions via Solid/Liquid Process: Use of Polymer-Modified Clay and Activated Carbons

**Cinzia Cristiani [1,*], Maurizio Bellotto [2], Giovanni Dotelli [1], Paola Gallo Stampino [1], Saverio Latorrata [1] and Elisabetta Finocchio [3,*]**

[1] Dipartimento di Chimica, Materiali e Ingegneria Chimica "Giulio Natta", Politecnico di Milano, Piazza Leonardo Da Vinci 32, 20133 Milan, Italy; giovanni.dotelli@polimi.it (G.D.); paola.gallo@polimi.it (P.G.S.); saverio.latorrata@polimi.it (S.L.)

[2] OPIGEO, SrL, Via dell'Industria 13, 36040 Grisignano di Zocco, Italy; maurizio.bellotto@opigeo.eu

[3] Dipartimento di Ingegneria Civile, Chimica e Ambientale, Università di Genova, Via all'Opera Pia 15, 16145 Genova, Italy

* Correspondence: cinzia.cristiani@polimi.it (C.C.); elisabetta.finocchio@unige.it (E.F.); Tel.: +39-02-23993248 (C.C.); +39-+39-010-3352919 (E.F.)

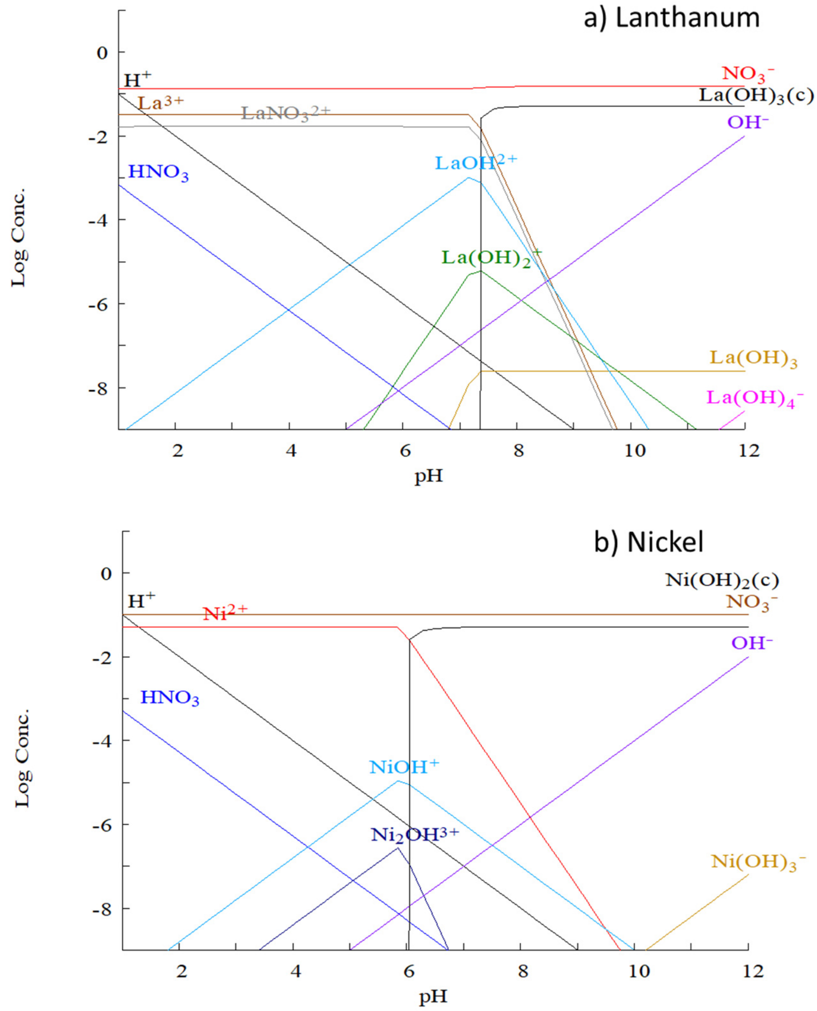

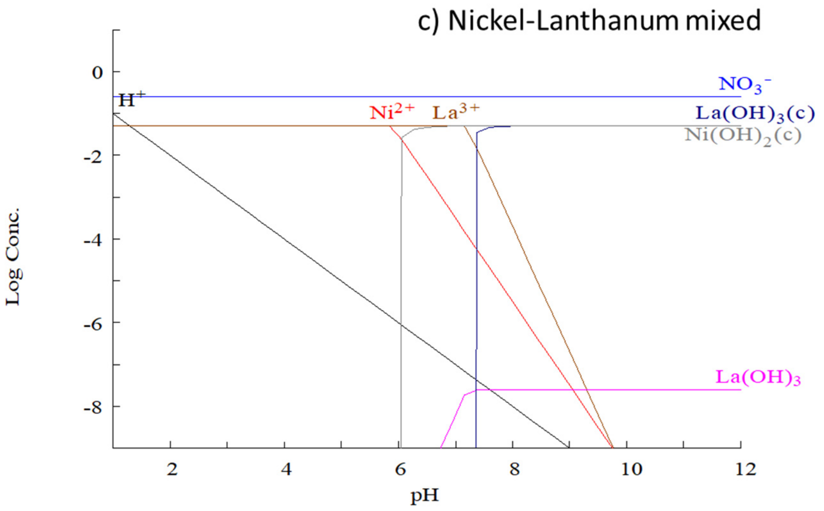


**Figure S1.** MEDUSA® plot of (**a**) Lanthanum single-ion solution, (**b**) Nickel single ion solution and (**c**) Nickel-Lanthanum mixed solution.

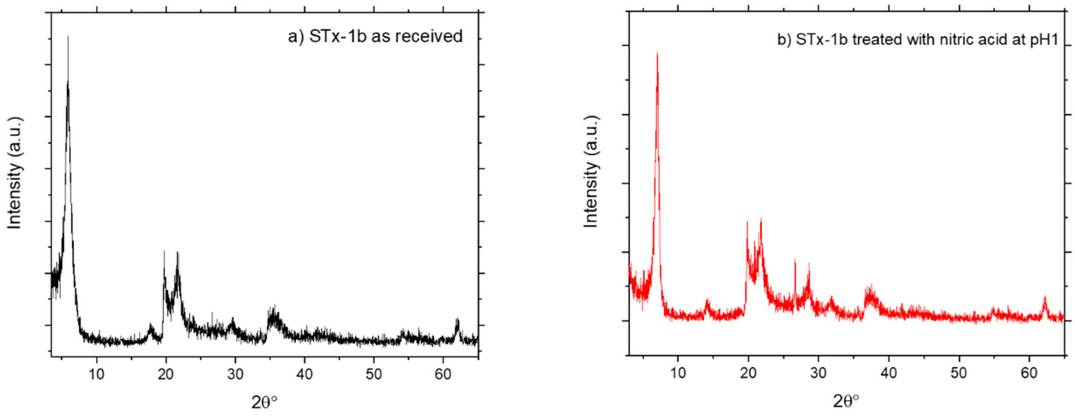


**Figure S2.** XRD patterns of (**a**) STx as received and (b) STx after contact with $HNO_3$ at pH = 1.
